# Antidiabetic potential of *Lysiphyllum strychnifolium* (Craib) A. Schmitz compounds in human intestinal epithelial Caco-2 cells and molecular docking-based approaches

**DOI:** 10.1186/s12906-022-03706-x

**Published:** 2022-09-05

**Authors:** Kunwadee Noonong, Kanta Pranweerapaiboon, Kulathida Chaithirayanon, Kantamat Surayarn, Phicharinee Ditracha, Narin Changklungmoa, Pornanan Kueakhai, Poonsit Hiransai, Kingkan Bunluepuech

**Affiliations:** 1 School of Allied Health Sciences, Walailak University, Nakhonsithammarat, Thailand; 2 Research Excellence Center for Innovation and Health Product, Walailak University, Nakhonsithammarat, Thailand; 3 Chulabhorn International College of Medicine, Thammasat University, Pathumthani, Thailand; 4 Department of Anatomy, Faculty of Science, Mahidol University, Bangkok, Thailand; 5 Faculty of Allied Health Sciences, Burapha University, Chonburi, Thailand; 6 School of Medicine, Walailak University, Nakhonsithammarat, Thailand

**Keywords:** *L. strychnifolium*, α-amylase, α-glucosidase, Caco-2 cell, Glucose transporters

## Abstract

**Background:**

*Lysiphyllum strychnifolium* (Craib) A. Schmitz, a traditional Thai medicinal plant, is mainly composed of polyphenols and flavonoids and exhibits several pharmacological activities, including antioxidant, anticancer, antimicrobial, and antidiabetic activities. However, the mechanism by which pure compounds from *L. strychnifolium* inhibit glucose catalysis in the small intestine and their effect on the glucose transporter remain unknown.

**Methods:**

The objectives of this research were to examine the effect of 3,5,7-trihydroxychromone-3-*O*-𝛼-L-rhamnopyranoside (compound 1) and 3,5,7,3’,5’-pentahydroxy-flavanonol-3-*O*-𝛼-L-rhamnopyranoside (compound 2) on the inhibition of α-amylase and α-glucosidase, as well as glucose transporters, such as sodium-glucose cotransporter 1 (SGLT1), glucose transporter 2 (GLUT2), and glucose transporter 5 (GLUT5), using Caco-2 cells as a model of human intestinal epithelial cells. Additionally, the binding affinity and interaction patterns of compounds against two receptor proteins (SGLT1 and GLUT2) were determined for the first time utilizing a molecular docking approach.

**Results:**

In the α-amylase inhibition assay, a concentration-dependent inhibitory response was observed against the enzyme. The results indicated that compound 1 inhibited α-amylase activity in a manner similar to that of acarbose (which exhibit IC_50_ values of 3.32 ± 0.30 µg/mL and 2.86 ± 0.10 µg/mL, respectively) in addition to a moderate inhibitory effect for compound 2 (IC_50_ = 10.15 ± 0.53 µg/mL). Interestingly, compounds 1 and 2 significantly inhibited α-glucosidase and exhibited better inhibition than that of acarbose, with IC_50_ values of 5.35 ± 1.66 µg/mL, 510.15 ± 1.46 µg/mL, and 736.93 ± 7.02 µg/mL, respectively. Additionally, α-glucosidase activity in the supernatant of the Caco-2 cell monolayer was observed. In comparison to acarbose, compounds 1 and 2 inhibited α-glucosidase activity more effectively in Caco-2 cells without cytotoxicity at a concentration of 62.5 µg/mL. Furthermore, the glucose uptake pathways mediated by SGLT1, GLUT2, and GLUT5- were downregulated in Caco-2 cells treated with compounds 1 and 2. Additionally, molecular modeling studies revealed that compounds 1 and 2 presented high binding activity with SGLT1 and GLUT2.

**Conclusion:**

In summary, our present study was the first to perform molecular docking with compounds present in *L. strychnifolium* extracts. Our findings indicated that compounds 1 and 2 reduced glucose uptake in Caco-2 cells by decreasing the expression of glucose transporter genes and inhibiting the binding sites of SGLT1 and GLUT2. Therefore, compounds 1 and 2 may be used as functional foods in dietary therapy for postprandial hyperglycemia modulation of type 2 diabetes.

## Background

Diabetes mellitus (DM) is a chronic metabolic disorder caused by insufficient insulin secretion, impaired insulin action, or a combination of the two, resulting in hyperglycemia. DM is among the most prevalent diseases in the twenty-first century, mainly due to a variety of lifestyle problems [1]. The chronic hyperglycemia of diabetes is a leading cause of blindness, kidney failure, heart attacks, stroke and lower limb amputation [2]. DM has become a global public health crisis, affecting millions of people worldwide, and the rise in the incidence of type 2 DM has been a concern worldwide. Over 90% of diabetic cases are type 2 DM, and with this disease, the pancreas produces insufficient or inefficient insulin, which is known as insulin resistance [3].

Carbohydrates are converted into glucose and enter the bloodstream in the small intestine. Alpha-amylase enzyme (α-amylase) and alpha-glucosidase (α-glucosidase) enzymes play an important role in catalyzing the hydrolysis of starch to glucose. Glucose cannot diffuse freely across the hydrophobic area of the lipid layer of the cell membrane and can only be absorbed and utilized in the small intestine via glucose transporters [4]. Numerous studies have established that glucose transporters are critical components of the glucose transport mechanism, most notably sodium-dependent glucose transporter 1 (SGLT1), epithelium glucose transporter 2 (GLUT2), and epithelial glucose transporter 5 (GLUT5) [5, 6]. Thus, the expression levels of SGLT1, GLUT2, GLUT5 and their associated proteins are critical in the process of glucose absorption in the small intestine.

Managing type-2 diabetes by conventional therapy involves inhibiting the degradation of dietary starch by glucosidases such as α-amylase and α-glucosidase [2]. Acarbose is an oral antidiabetic drug that potentially inhibits α-amylase and α-glucosidase enzymes. However, long-term use of these drugs involves a number of negative consequences, including bloating, indigestion, and liver toxicity [7]. Recently, herbs have been utilized as alternative agents to control blood glucose. *L. strychnifolium* is a reddish climbing plant that is found in montane tropical rainforests and mixed deciduous forests. The leaves, vines, and roots of *L. strychnifolium* have an astringent, sweet, and cold flavor. Additionally, *L. strychnifolium*, a traditional Thai medicinal plant, is widely used for detoxification purposes [8]. The main chemical components of *L. strychnifolium* are polyphenols and flavonoids, and *L. strychnifolium* exhibits several pharmacological activities, including antioxidant, anticancer, antibacterial, antiviral, and antidiabetic activities [9–11]. The water fraction of *L. strychnifolium* contains 3,5,7-trihydroxychromone-3-*O*-α-L-rhamnopyranoside and 3,5,7,3’,5’-pentahydroxy-flavanonol-3-*O*-𝛼-L-rhamnopyranoside, which inhibit α-glucosidase activity [12]. However, the mechanism by which *L. strychnifolium* extracts inhibit α-amylase activity and α-glucosidase in the small intestine and affects glucose transporters remains unknown.

Herein, we emphasized the antidiabetic effects of pure compounds, 3,5,7-trihydroxychromone-3-*O*-α-L-rhamnopyranoside (compound 1) and 3,5,7,3’,5’-pentahydroxy-flavanonol-3-*O*-𝛼-L-rhamnopyranoside (compound 2), that were isolated from *L. strychnifolium* for in vitro studies of α-amylase activity and α-glucosidase activity. Moreover, the effect of these compounds on glucose transporters in human intestinal epithelial Caco-2 cells was investigated. Additionally, the cytotoxicity and mRNA expression of genes involved in glucose uptake (SGLT1, GLUT2, and GLUT5) were determined using the MTT assay and RT‒PCR, respectively. Furthermore, we explored the compound expected to be responsible for the antidiabetic activity by performing molecular docking with the identified compounds toward the glucose transporter.

## Materials and methods

### Reagents

Culture medium DMEM (high glucose), DMEM (without glucose), fetal bovine serum, penicillin‒streptomycin and phosphate buffer solution were purchased from Invitrogen Corporation (Massachusetts, USA). 3-(4,5-Dimethylthiazol-2-yl)-2,5-diphenyl tetrazolium bromide (MTT), 1,1-diphenyl-2-picrylhydrazyl (DPPH), 2,2’-azinobis (3-ethylbenzothiaz oline-6-sulfonic acid) diammonium salt (ABTS), α-amylase, α-glucosidase, maltose and acarbose were purchased from Sigma‒Aldrich Corporation (St. Louis, MO, USA). Analytical grade chemicals and reagents were utilized in the studies.

### Plant materials

*L. strychnifolium* stems were collected in 2020 at the Suan Ya Thai Thongnoppakhun herbal garden in Chonburi Province. A voucher specimen (SKP 072021901) is currently being stored at the Herbarium of the Department of Pharmacognosy and Pharmaceutical Botany, Faculty of Pharmaceutical Sciences, Prince of Songkla University, Thailand.

### Preparation of *L. strychnifolium* extracts and their isolated compounds

*L. strychnifolium* (4.5 kg) was extracted twice at room temperature with EtOH (28 L). Under reduced pressure, a 993.5 g EtOH extract was obtained and maintained at 4 °C. The EtOH extract (993.5 g) was sequentially partitioned with hexane, dichloromethane, ethyl acetate, and water. The water and chloroform fractions that exhibited good activity in enzyme assays were selected to further isolate bioactive compounds. The 40 g water fraction was chromatographed in water, water/methanol, and methanol to yield 6 fractions (F1-F6). Fraction F6 (6.2 g) was separated by silica gel column chromatography using 10% methanol in ethyl acetate. Subfraction F6/3a (3.24 g) was purified by column chromatography on silica gel using 20% methanol in chloroform. Subfraction F6/2b was purified by silica gel column chromatography using 20% methanol in chloroform to extract 3,5,7-trihydroxychromone-3-*O*-𝛼-L-rhamnopyranoside (compound 1) and 3,5,7,3’,5’-pentahydroxy-flavanonol-3-*O*-𝛼-L-rhamnopyranoside (compound 2). Pure chemicals (compounds 1 and 2) were isolated by chromatography, and their spectroscopic data were compared to earlier reports [11, 12].

### Determination of ABTS scavenging assay

The method by Kumar, Sanjiv et al. [13] was adopted for the ABTS (2,2′-azino-bis-(3-ethylbenzothiazoline-6-sulfonate) assay with slight modifications. Briefly, 1:44 (v/v) ABTS solution (100 µl) and extract solution (100 µl) were added to a 96-well microplate and thoroughly mixed. The absorbance was measured at 734 nm after a 6-minute incubation time at room temperature. The ABTS scavenging activity was calculated using the equation below and was expressed as 50% effective concentration (EC_50_).

$$\%\;\mathrm{ABTS}\;\mathrm{scavenging}\;\mathrm{activity}\;=\left[\frac{{\left(O.D\right)}_{control}-{\left(O.D\right)}_{sample}}{{\left(O.D\right)}_{control}}\right]\times100$$

### Determination of the DPPH radical scavenging assay

The method by Kumar, Sanjiv et al. [13] was adopted for DPPH (2,2-diphenyl-1-picrylhydrazyl) assays with slight modifications. Briefly, DPPH solution (100 µl) and extract solution (100 µl) were added to a 96-well microplate and thoroughly mixed. The absorbance was measured at 517 nm after a 30-minute incubation time at room temperature. The DPPH scavenging activity was calculated using the equation below and was expressed as the 50% effective concentration (EC_50_).

$$\%\;\mathrm{DPPH}\;\mathrm{scavenging}\;\mathrm{activity}\;=\left[\frac{{\left(O.D\right)}_{control}-{\left(O.D\right)}_{sample}}{{\left(O.D\right)}_{control}}\right]\times100$$

### Determination of the α-amylase inhibition assay

The method by Poovitha S and Parani M [14] was adopted for the α-amylase inhibition assay with slight modifications. Each 96-well microplate was filled with extract solutions (50 µl) and α-amylase solution, 0.5 mg/mL (25 µl). The reaction mixtures were incubated for 10 min at room temperature. Then, a 1% starch solution (50 µl) in 20 mM sodium phosphate buffer (pH 6.9 with 6 mM sodium chloride) was added and incubated for 10 min at room temperature. To stop the reaction after incubation, 100 µl of dinitro salicylic acid color reagent was added, and the microplate was then heated in a boiling water bath for 10 min before being cooled at room temperature. The absorbance of the reaction mixture was measured at 540 nm. The percent inhibition of all samples was calculated using the following equation and was expressed as the 50% inhibitory concentration (IC_50_).

$$\%\;\mathrm{inhibition}\;=\left[\frac{{\left(O.D\right)}_{control}-{\left(O.D\right)}_{sample}}{{\left(O.D\right)}_{control}}\right]\times100$$

### Determination of the α-glucosidase inhibition assay

The method by Poovitha S and Parani M [14] was adopted for the α-amylase inhibition assay with slight modifications. Each 96-well microplate was filled with sample solutions (25 µl) and α-glucosidase solution, 0.5 unit/mL (25 µl). The reaction mixtures were incubated for 10 min at 37 °C. Then, pNPG (25 µl) in 20 mM sodium phosphate buffer (pH 6.8 with 6 mM sodium chloride) was added and incubated for 30 min at 37 °C. To stop the reaction after incubation, 100 µl of 0.2 M Na_2_CO_3_ reagent was added. The absorbance of the reaction mixture was measured at 405 nm. The percent inhibition of all samples was calculated using the following equation and was expressed as the 50% inhibitory concentration (IC_50_).

$$\%\;\mathrm{inhibition}\;=\left[\frac{{\left(O.D\right)}_{control}-{\left(O.D\right)}_{sample}}{{\left(O.D\right)}_{control}}\right]\times100$$

### Cell culture

Caco-2 cells were obtained from the American Type Culture Collection (ATCC). The cells were cultured in DMEM with 10% FBS and 1% penicillin–streptomycin solution and were maintained at 37 °C in a humidified environment of 95% air and 5% CO_2_. For the viability test, the Caco-2 cells were seeded on 96-well plates, and 24-well Transwell plates were used to determine α-glucosidase inhibition. The cells differentiated after 21 days and exhibited intestinal microvillus membrane hydrolases [15]. Fresh media was supplied to the cells every two days during this period.

### Cell viability assay

Caco-2 cells were obtained from the American Type Culture Collection (ATCC). The cells were grown in complete medium (DMEM with 10% FBS, 100 units/mL penicillin, and 100 µg/mL streptomycin) and plated at a density of 10,000 cells per well in 96-well plates for 21 days. Then, the cells were treated with extracts at concentrations ranging from 0 to 500 g/ml for another 24 h. DMSO (0.1%) was added to the control group. The MTT test was used to measure cell viability after treatment. In a 96-well plate, 100 µl of MTT at a concentration of 0.5 mg/ml was applied to each well. The MTT solution was withdrawn after 3 h of incubation at 37 °C, and formazan crystals were dissolved in 100 µl of DMSO. At 570 nm, the purple color that was generated was detected spectrophotometrically (with a reference OD of 690) [16]. Prism 6 software was used to calculate cell viability. The appropriate concentration of extracts was chosen for future experiments.

### Inhibition of α-glucosidase activity assay in Caco-2 cells

Caco-2 cells were seeded in 24-transwell plates at a density of 10,000 cells per well for 21 days. The cells were washed in 1X PBS after the culture media was removed. The upper chamber was then filled with compounds/acarbose and 28 mM maltose in glucose-free media (300 µl) for 24 h overnight. At the same time, the lower chamber was filled with glucose-free medium (500 µl). Then, 50 µl of culture media from the upper chamber was taken and measured by the glucose oxidase method [17]. The commercial α-glucosidase inhibitor acarbose was used as a positive control to verify the culture system.

### Detection of the transcription of SGLT1, GLUT2, and GLUT5 mRNA in Caco-2 cells

RNA was extracted from Caco-2 cells using an RNA extraction kit (QIAGEN, Hilden, Germany). Reverse transcription was performed with SuperScript III Reverse Transcriptionase (Thermo Fisher Scientific, Waltham, USA). cDNA was synthesized from 2 µg of total RNA according to the manufacturer’s instructions. Then, the cDNA was maintained at − 20°C for further analysis by real-time PCR. The specific primers used in the experiment were as follows: SGLT1, 5’-TGGCATCAACGCTGTCTTCT-3’ (forward) and 3’-AGCCAATGGTGGCATACACA-5’ (reverse); GLUT2, 5’-CTGCCGCTGAGAAGATTAGAC-3’ (forward) and 3’-CAGGTCTTGTGTGAGTGTGG-5’ (reverse); GLUT5, 5’- TCCATTTGGAGGGTTTATCG-3’ (forward) and 3’-AACAGCAAGGCCCCCTTTT-5’ (reverse). All conditions were performed in triplicate with 3 independent experiments. The real-time qPCR mixture (10 µL) comprised 1 µL of DNA polymerase, template DNA (25 ng), 1 µL of DEPC, and 1.2 µL of forward and reverse primers. Thermal cycling (Applied Biosystems, Waltham, USA) consisted of (denaturation 95 °C for 20 s, annealing 63.3 °C for 30 s, and extension 72 °C for 20 s, 40 cycles. The gene expression data were normalized to the internal control gene GAPDH and then compared using comparative delta-delta Ct.

### Molecular docking and chemical visualization

Molecular docking was performed to illustrate the molecular interaction between the two major compounds derived from *L. strychnifolium*. 3,5,7-trihydroxychromone-3-*O*-𝛼-L-rhamnopyranoside (compound 1), 3,5,7,3’,5’-pentahydroxy-flavanonol-3-*O*-𝛼-L-rhamnopyranoside (compound 2), acarbose, and the glucose transporter membrane (SGLT1 and GLUT2). The crystalized structures of SGLT1 and GLUT2 were downloaded from the RCSB protein data bank as PDB IDs 2XQ2 and 4ZWB, respectively [18, 19]. All 3D structures of ligands and proteins were generated and optimized by LigandScout 4.4 Advanced (Intel: Ligand GmbH, Vienna, Austria) [20]. The binding sites of SGLT1 and GLUT2 were predicted to be cocrystalized by LigandScout 4.4 and served as the basis of the *in silico* experiments. The binding poses, interactions, and binding affinities of *acarbose*, compounds and SGLT1 and GLUT2 were determined by using the built-in AutoDock Vina 1.1 module of LigandScout 4.4 advanced. The native ligand was redocked into its binding site to validate the methods. The redocking RMSD values were 0.60 and 0.00 Å, respectively.

### Statistical analysis

The results are presented as the means ± SEMs and were analyzed by using GraphPad Prism 5.0 (GraphPad Software). Statistical comparisons were determined by one-way ANOVA, followed by two-tailed Student’s t test. Statistical significance was defined as a *p* value of less than 0.05.

## Results

### Compounds exhibiting antioxidant activity

Two methods were used to evaluate the antioxidant activities, including the DPPH radical scavenging activity and ABTS radical scavenging activity. These assays offer a redox-functioning proton ion for unstable free radicals and play a critical role in stabilizing detrimental free radicals in the human body. The scavenging capacity of the compounds is indicated by the degree of reduction in absorbance measurement. Thus, we examined the free radical scavenging activity of compound 1 and compound 2 in comparison to ascorbic acid (positive control), as shown in Table 1. Compounds 1 and 2 exhibited similar antioxidant activity in the ABTS assay, with EC_50_ (the concentration that causes 50% inhibition) values of 10.26 ± 0.20 µM and 14.40 ± 0.10 µM, respectively, when compared to that of ascorbic acid (25.10 ± 0.19 µM). In addition, the EC_50_ values of compounds 1 and 2 were 5.22 ± 0.20 µM and 5.29 ± 0.12 µM, respectively, as determined by the DPPH assay. All compounds demonstrated significantly higher antioxidant activity than that of positive controls.

**Table 1:** EC_50_ values for the antioxidant activity of compounds 1 and 2 measured with ABTS and DPPH assays. Each value represents the mean ± SEM, *** P < 0.001

Samples	ABTS scavenging assay (µM)	DPPH radical scavenging assay (µM)
**Compound 1**	10.26 ± 0.20 ^***^	5.22 ± 0.20 ^***^
**Compound 2**	14.40 ± 0.10 ^***^	5.29 ± 0.12 ^***^
**Ascorbic acid (Positive control)**	25.10 ± 0.19	30.38 ± 0.18

### Compounds that inhibited α-amylase and α-glucosidase activity

**Fig. 1:**
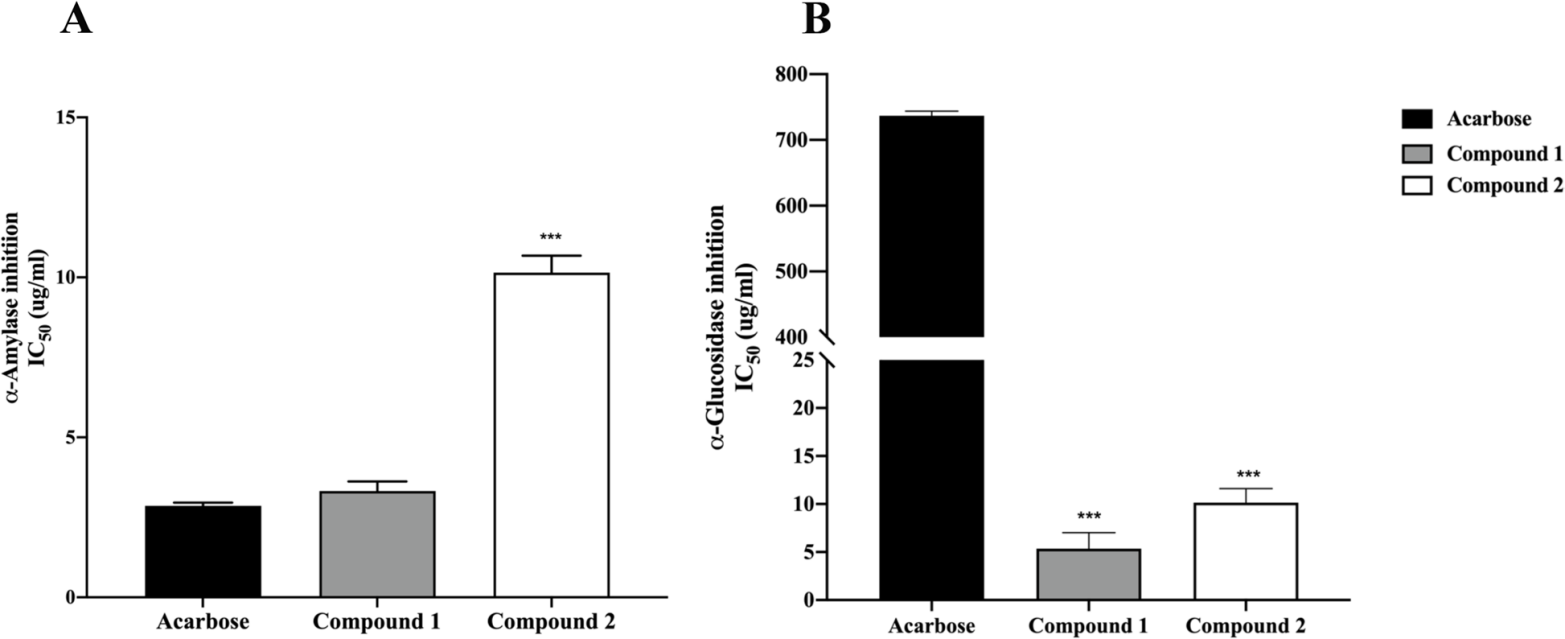
IC_50_ values of compound 1, compound 2, and acarbose in α-amylase inhibition (**A**) and α-glucosidase inhibition (**B**). Each value represents the mean ± SEM, *** P < 0.001

The inhibition of α-amylase activity by compounds 1 and 2 and acarbose was found to be dose dependent. Compound 1 exhibited similar inhibition of α-amylase activity to that of acarbose, with IC_50_ values of 7.37 ± 0.30 µM and 4.43 ± 0.10 µM, respectively. In addition, compound 2 showed less efficient inhibition of α-amylase activity than that of acarbose, with IC_50_ values of 29.82 ± 0.53 µM (Fig. 1A). On the other hand, compounds 1 and 2 showed significantly greater inhibition of α-glucosidase than that of acarbose, with IC_50_ values of 11.88 ± 1.66 µM, 29.83 ± 1.46 µM, and 1141.46 ± 7.02 µM, respectively (Fig. 1B).

### Compounds that inhibited α-glucosidase activity in Caco-2 cells

**Fig. 2:**
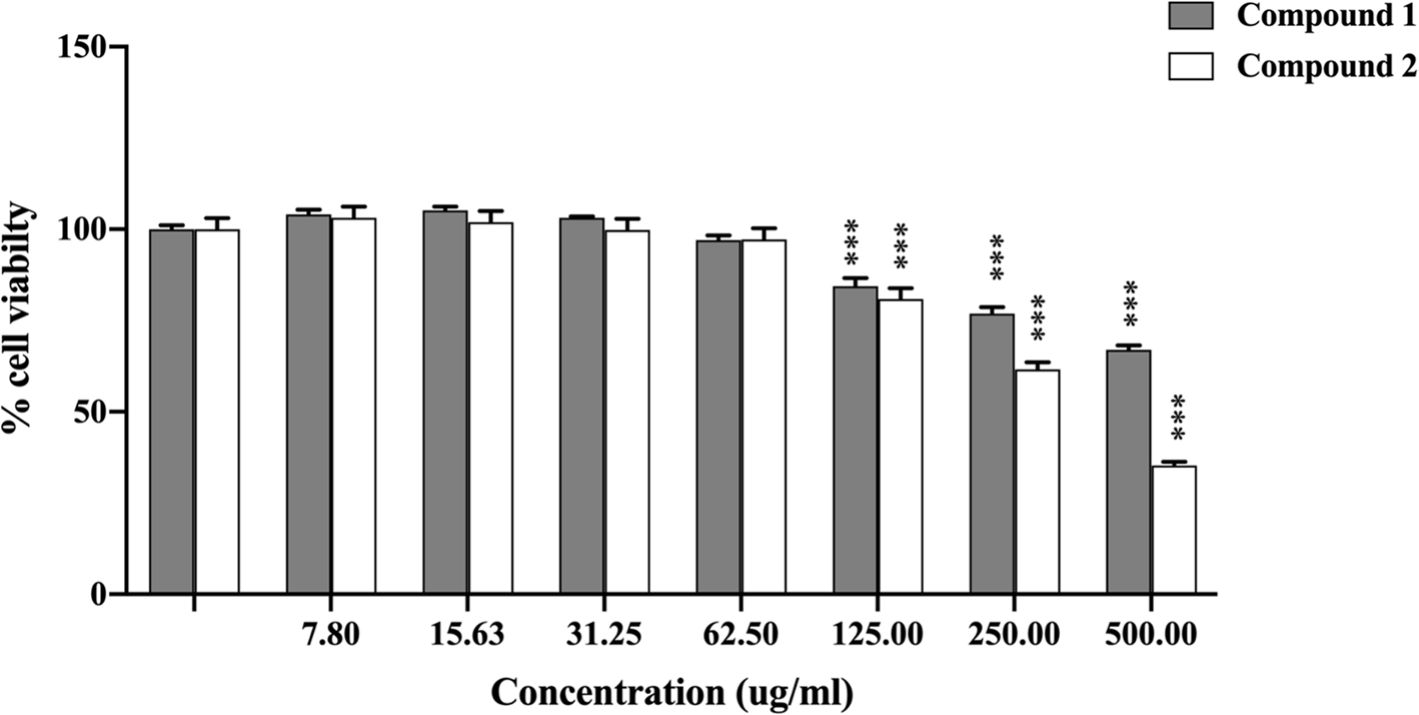
Caco-2 cell viability in the presence of compounds 1 and 2 (ranging from 0-500 ug/ml) using the MTT assay. Each value represents the mean ± SEM, *** P < 0.001

**Fig. 3:**
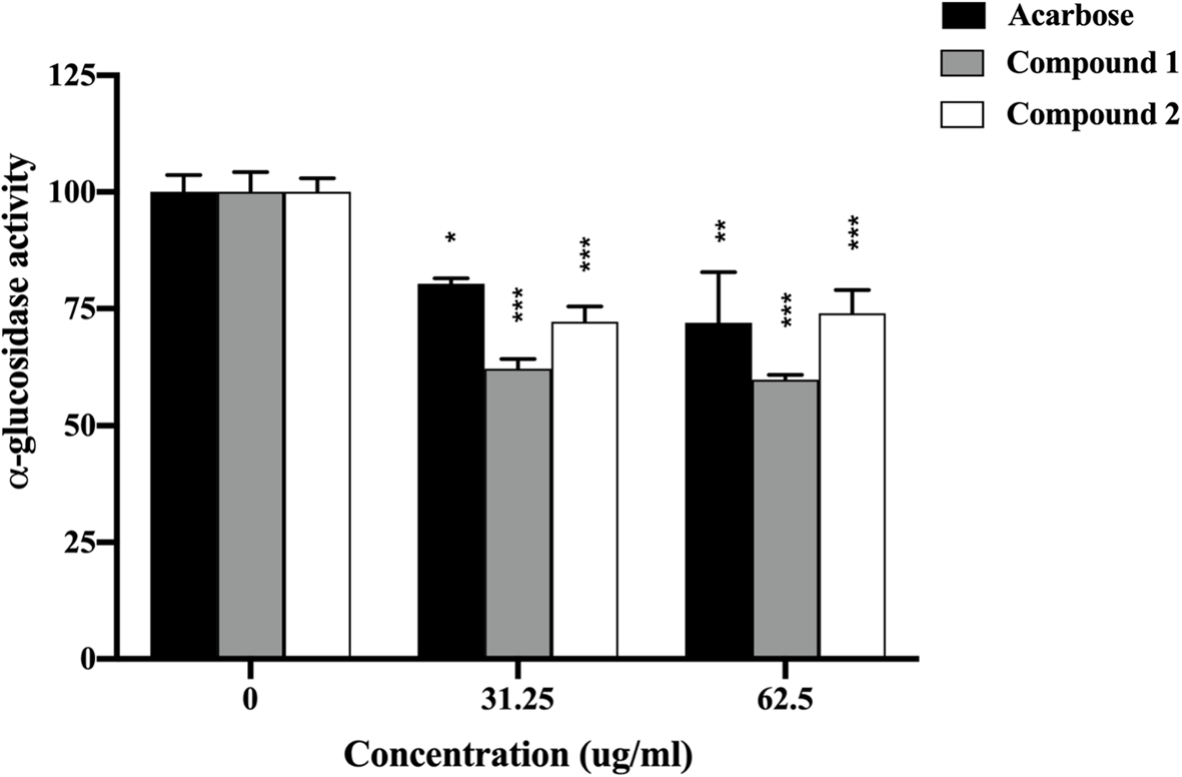
α-Glucosidase activity with the Caco-2 monolayer in the presence of compounds 1, 2, and acarbose (31.25 ug/ml and 62.50 ug/ml). Each value represents the mean ± SEM, * P < 0.05, ** P < 0.01, *** P < 0.001

Caco-2 cells have been widely used as a culture model for human intestinal cells in studies to determine the inhibitory activity of α-glucosidase [21]. To determine whether compounds 1 and 2 inhibit α-glucosidase activity in Caco-2 cells, we first determined their viability using the MTT assay. The results indicated that concentrations ranging from 0 to 62.50 ug/ml were not cytotoxic to Caco-2 cells (Fig. 2). As a result, concentrations less than 62.50 ug/ml were chosen for further investigation. Next, we determined the efficiency of which compounds 1 and 2 inhibited α-glucosidase activity using the Caco-2 monolayer. Compounds 1 and 2 were evaluated for their inhibitory effect on the apical sides of the Caco-2 monolayer. As shown in Fig. 3, cells treated with compounds 1, 2, and acarbose showed significantly decreased α-glucosidase activity compared with that of untreated cells, with values of 59.75%, 73.90%, and 71.95%, respectively. Compound 1 showed the highest effect on the inhibition of α-glucosidase activity when measured on the apical sides of the Caco-2 monolayer.

### Effect of the compounds on the transcription of SGLT1 and GULT2 mRNA in Caco-2 cells

**Fig. 4:**
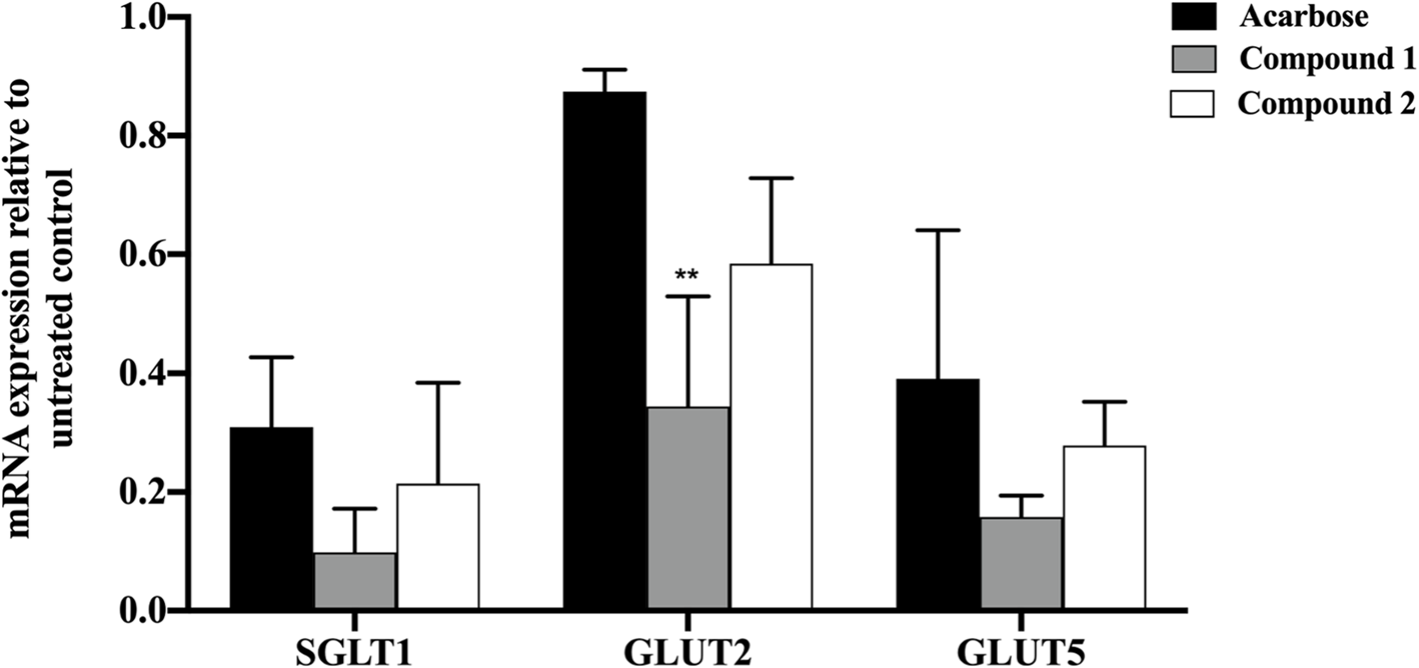
Effect of compounds 1, 2, and acarbose (62.50 µg/ml) on the mRNA expression of SGLT1, GLUT2, and GLUT5 in Caco-2 cells. The results are expressed as fold changes compared to the housekeeping gene (GAPDH). Each value represents the mean ± SEM, ** P < 0.01

To explore the mechanism of compounds 1 and 2 in promoting the absorption of Caco-2 glucose, the transcription of SGLT1, GULT2, and GLUT5 signaling pathway mRNA in Caco-2 cells was explored by using RT‒PCR. Compounds 1, 2 and acarbose downregulated the transcription of the SGLT1, GULT2, and GLUT5 genes, as illustrated in Fig. 4. Additionally, compared to acarbose, compound 1 was significantly downregulated in the GULT2 gene. Therefore, these results suggest that compounds 1 and 2 could decrease glucose uptake in Caco-2 cells by downregulating the mRNA transcription levels of SGLT1, GULT2, and GLUT5.

### Molecular docking

**Fig. 5:**
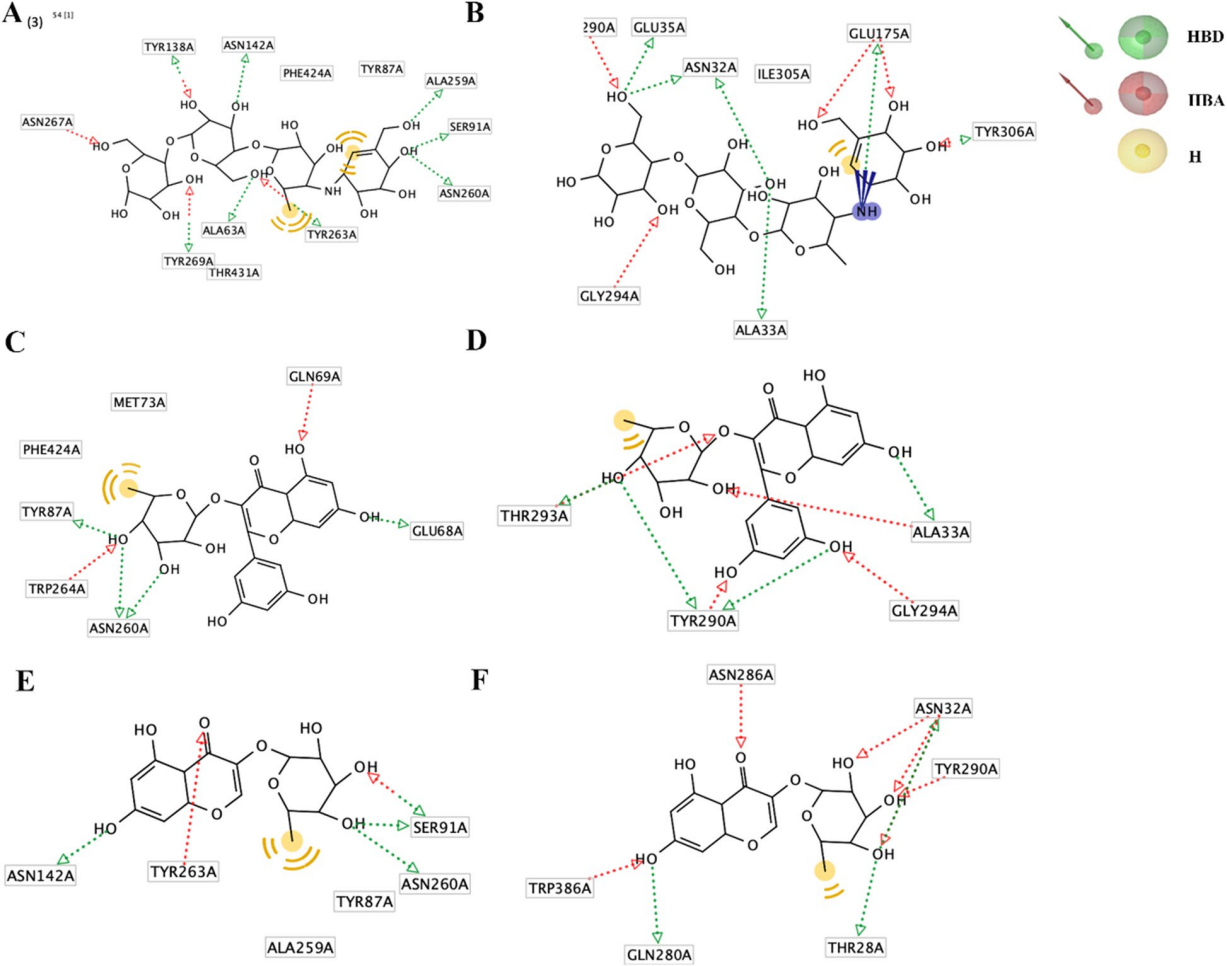
Docking of compounds 1, 2, and acarbose with SGLT1 and GLUT2. **A**, **C**, and **E** represent interactions of acarbose, compounds 1, and 2 with SGLT1, respectively. **B**, **D**, and **F** represent the interactions of acarbose, compounds 1, and 2 with GLUT2, respectively

A docking study was performed to reveal the antidiabetic activity of compounds 1 and 2 against the glucose transporter and to explore the compounds that were expected to be responsible for their binding modes to the glucose transporter (SGLT1 and GLUT2) in the human small intestine (Table 2; Fig. 5A-F). The interactions of acarbose, compounds 1, and 2 with SGLT1 are summarized in Fig. 5A, C and E, respectively. The interactions of acarbose, compounds 1, and 2 with GLUT2 are summarized in Fig. 5B, C and F, respectively. The binding pocket of the SGLT1 receptor protein contains Asn267, Tyr138, Tyr263, Ser368, and Thr431 as the main interacting amino acids. Compound 1 showed the best interactions (binding score: -24.30) with the SGLT1 receptor, and Tyr87, Asn260, Glu68, Gln69, Trp264, Met73, and Phe424 were found to be the leading interactive residues in these interactions (Table 2; Fig. 5C). Acarbose and compound 2 obtained docking scores of -23.30 and − 21.00, respectively. Similarly, the library of compounds 1, 2 and acarbose were also docked against GLUT2 receptor proteins. Compound 1 interacted with Thr293, Thr290, Gly294, Ala33, Thr293, Thr290, Gly294, and Ala33 of the GLUT2 receptor protein and exhibited a binding score of -17.90 (Table 2; Fig. 5D). Additionally, compound 2 showed the best interactions (binding score: -20.70) with the GLUT2 receptor.

**Table 2:** Chemical interactions of acarbose, compound 1, compound 2 and the glucose transporter membrane (SGLT1 and GLUT2). Hydrogen bond donor (HBD), hydrogen bond acceptor (HBA) and hydrophobic interaction (H)

Targets	Compound	Binding affinities (kcal/mol)	Residues
SGLT1 (2xq2)	Acarbose	-23.30	HBD (TYR263, ASN260, SER91, ALA259, ASN 142, TYR138) HBA (ASN267, TYR269, TYR263, TYR138) H (THR431, ILE427, TYR87, PHE424)
	Compound 1	-24.30	HBD (TYR87, ASN260, GLU68) HBA (GLN69, TRP264) H (MET73, PHE424)
	Compound 2	-21.00	HBD (ASN142, ASN260, SER91) HBA (TRP263, SER91) H (ALA259, TYR87)
GLUT2 homolog (4ZWB)	Acarbose	-17.50	HBD (GLU35, ASN32, ALA33, GLU175, TYR306) HBA (TYR290, GLY294, GLU175, TYR306) H (ILE305) IA (GLU175)
	Compound 1	-17.90 (same position with acarbose)	HBD (THR293, THR290, GLY294, ALA33) HBA (THR293, THR290, GLY294, ALA33)
	Compound 2	-20.70 (same position with acarbose)	HBD (GLN280, THR28, ASN32) HBA (ASN286, TRP386, ASN32, TYR290) H (THR28)

## Discussion

Type 2 DM is a metabolic disorder characterized by prolonged periods of elevated blood glucose (hyperglycemia), which results in insulin resistance in peripheral tissues or impaired insulin production by pancreatic B cells [1, 3]. Controlling postprandial glycemia may be a strategy for preventing the development of DM2 and other complications associated with cardiovascular disease, macular degeneration, kidney disease, etc. [2, 22]. There are currently several oral antihyperglycemic medicines available and each exhibits a unique mode of action for maintaining normal glycemia. These medications include inhibitors of intestinal α-glucosidases, which delay glucose absorption in the intestine; metformin, which inhibits hepatic gluconeogenesis; and sodium/glucose cotransporter 2 (SGLT-2) inhibitors, which impair glucose reuptake [23]. Acarbose, α-amylase inhibitor and α-glucosidase inhibitors prevent carbohydrates from being digested, resulting in improved glycemic control. However, acarbose cannot be used as a long-term treatment method because side effects occur over the treatment period and cause abdominal bloating, cramping, and flatulence [7, 24]. According to the World Health Organization (WHO), herbal medicine continues to be the most widely used primary health care modality, particularly in developing countries, due to its cultural acceptability, compatibility with the human body, and absence of adverse effects. As a result, the exploration of natural sources for new inhibitors is necessary [25, 26].

Here, we examined the effects of 3,5,7-trihydroxychromone-3-*O*-𝛼-L-rhamnopyranoside (compound 1) and 3,5,7,3’,5’-pentahydroxy-flavanonol-3-*O*-𝛼-L-rhamnopyranoside (compound 2) on α-amylase inhibition and α-glucosidase inhibitory activity. We considered that compounds 1 and 2 are involved in the degradation of polysaccharides into disaccharides, in which compound 1 exhibited strong inhibition of both α-amylase and α-glucosidase. Comparison of the half maximal inhibitory concentration (IC_50_) values revealed that the α-amylase inhibitory activity of compound 1 (IC_50_ 3.32 µg/mL) was similar to that of acarbose (IC_50_ 2.86 µg/mL). In addition, the IC_50_ values of compound 1 (IC_50_ 5.35 µg/mL) inhibited α-glucosidase activity by approximately 133% compared with that of acarbose (IC_50_ 736.93 µg/mL). In comparison to previous studies, Ficus racemosa Linn. fruit ethanolic extract showed an inhibitory effect of α-amylase with an IC_50_ value of 7.44 µg/mL [27]. The IC_50_ values of *Ficus carica* L. in the inhibition of α-amylase and α-glucosidase were 315.89 ± 3.83 µg/mL and 255.57 ± 36.46 µg/mL, respectively [28].

α-Glucosidase degrades disaccharides into monosaccharides, such as glucose, in the small intestine prior to absorption. According to cell lines, Caco-2 cells express the same morphological characteristics and most of the functional properties of terminally differentiated small intestinal enterocytes and express brush-border enzymes, such as α-glucosidase [15, 21, 29]. Therefore, we examined the inhibitory effects of compounds 1 and 2 on α-glucosidase activity in Caco-2 cells. In our investigation, intact cells grown in Transwells were employed to stimulate enzymatic hydrolysis without harming the cell monolayer, which is a physiologically realistic method [29]. Compounds 1 and 2 showed significantly and dose-dependently inhibited α-glucosidase activity when measured on the apical sides of the Caco-2 monolayer. Thus, compounds 1 and 2 not only inhibited α-amylase and α-glucosidase activity but also affected α-glucosidase activity in Caco-2 cells.

Glucose uptake in the small intestine is most notably mediated by sodium-dependent glucose transporter 1 (SGLT1), epithelium glucose transporter 2 (GLUT2), and epithelial glucose transporter 5 (GLUT5) [5, 6]. Thus, the expression levels of SGLT1, GLUT2, GLUT5 and their associated proteins are critical in the process of glucose absorption in the small intestine. SGLT1 utilizes the energy of the sodium electrochemical gradient that is maintained by basolateral Na-K ATPase activity. The second transport step at the basolateral membrane is carried out by GLUT2. GLUT2 and GLUT5, which are high-capacity, low-affinity transporters and are now involved in the major pathways of glucose absorption [5, 6, 30]. Then, we examined the effect of compounds 1 and 2 on the glucose uptake pathways mediated by SGLT1, GLUT2, and GLUT5 by using Caco-2 cells. Compounds 1, compound 2, and acarbose downregulated SGLT1, GLUT2, and GLUT5. Additionally, molecular modeling studies revealed that compounds 1 and 2 effectively interacted with the SGLT1 and GLUT2 binding sites. Altogether, it might be implied that compounds 1 and 2 are involved in the inhibition of SGLT1, GLUT2, and GLUT5 and consequently reduce glucose absorption from the intestine. To reveal the antidiabetic activity of compounds 1 and 2 against the glucose transporter, a docking study was performed to explore the compounds that were expected to be responsible for their binding modes to the glucose transporter (SGLT1 and GLUT2) in the human small intestine. Compounds 1 and 2 effectively interacted with the SGLT1 and GLUT2 binding sites. The docking data demonstrated that both compounds could form hydrogen bonds with TRP263 and TRP264 of SGLT1. Disrupting these positions induces a conformational change in SGLT1, which allows water to penetrate and prevents sugar from binding to the binding site [18]. In addition, the main mechanism used to interfere with the activity of GLUT2 is regulation of the conformation. A previous study suggested that binding at TYR290 and ASN286 affects the flexibility of the helix [19]. The molecular docking revealed the compounds that possibly bond with TYR290 and ASN286. This may indicate that the antidiabetic activity of compound 1 and compound 2 may also target the binding site of GLUT2. However, confirming the *in silico* results is necessary to ensure that the identification of efficacy is accurate, as there is a possibility of false positive and negative results, which is one of the limitations of the study.

Oxidative stress plays a role in the pathogenesis of type 2 diabetes and its complications. Increased levels of free radical and oxidative stress are associated with lipid peroxidation, nonenzymatic glycation of proteins, and glucose oxidation, all of which contribute to the development of diabetes and its complications, including coronary artery disease, nephropathy, retinopathy, and neuropathy [31]. We found that compounds 1 and 2 exhibited a high level of antioxidant activity, suggesting that ameliorating oxidative stress might be an effective strategy for reducing diabetic complications.

**Fig. 6:**
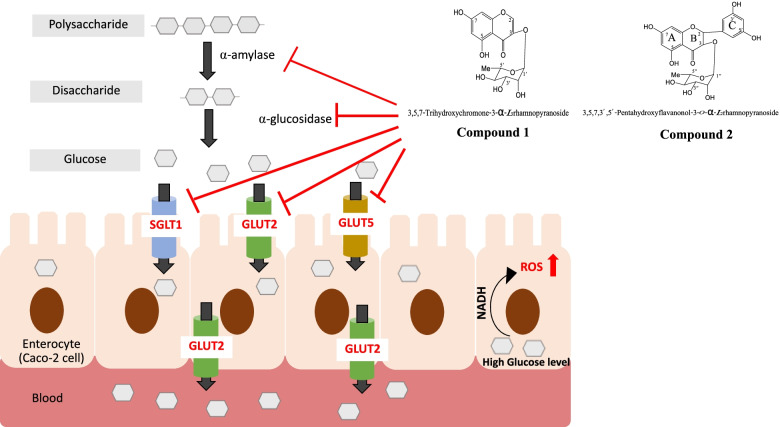
The proposed mechanisms by which 3,5,7-trihydroxychromone-3-*O*-α-L-rhamnopyranoside (compound 1) and 3,5,7,3’,5’-pentahydroxy-flavanonol-3-*O*-𝛼-L-rhamnopyranoside (compound 2) inhibit glucose catalysis in enterocytes of the small intestine and their effect on the glucose transporter SGLT1, GLUT2, and GLUT5

## Conclusion

Our findings demonstrate that 3,5,7-trihydroxychromone-3-*O*-α-L-rhamnopyranoside and 3,5,7,3’,5’-pentahydroxy-flavanonol-3-*O*-𝛼-L-rhamnopyranoside derived from *L. strychnifolium* have antioxidant and antidiabetic effects, as evidenced by α-amylase inhibition activities, α-glucosidase inhibition activities, and decreased glucose absorption in enterocytes of the small intestine by suppressing the gene expression of glucose transporters and inhibiting the binding sites of SGLT1 and GLUT2 (Fig. 6). Therefore, 3,5,7-trihydroxychromone-3-*O*-α-L-rhamnopyranoside and 3,5,7,3’,5’-pentahydroxy-flavanonol-3-*O*-𝛼-L-rhamnopyranoside may be used as functional foods in dietary therapy for postprandial hyperglycemia modulation of type 2 diabetes without the side effects associated with acarbose treatments.

## Data Availability

All data generated or analysed during this study are included in this published article.

## Abbreviations

*L. strychnifolium*: *Lysiphyllum strychnifolium (Craib) A. Schmitz*
compound 1: 3,5,7-trihydroxychromone-3-*O*-𝛼-L-rhamnopyranoside
compound 2: 3,5,7,3’,5’-pentahydroxy-flavanonol-3-*O*-𝛼-L-rhamnopyranoside
SGLT1: sodium-glucose cotransporter 1
GLUT2: glucose transporter 2
GLUT5: glucose transporter 5
OD: optical density
EC_50_: 50% effective concentration
IC50: 50% inhibitory concentration
